# The Relationship between Self-Perceived Health and Physical Activity in the Mental Health of Korean Cancer Survivors

**DOI:** 10.3390/healthcare11111549

**Published:** 2023-05-25

**Authors:** Sungjung Kwak, Jieun Shin, Jong-Yeup Kim

**Affiliations:** 1 Department of Nursing, Howon University, Gunsan 54058, Republic of Korea; sungjunglob@hanmail.net; 2 Department of Biomedical Informatics, College of Medicine, Konyang University, Daejeon 35365, Republic of Korea; 3 Healthcare Data Science Center, Konyang University Hospital Daejeon, Daejeon 35365, Republic of Korea

**Keywords:** self-perceived health, physical activity, mental health, cancer survivor, NHANES

## Abstract

The purpose of this study was to investigate the relationship between self-perceived health and physical activity on the mental health of cancer survivors using the 2014, 2016, 2018, 2020 National Health and Nutrition Examination Survey (NHANES) data. The study included 378 participants aged 19 years or older who had been diagnosed with cancer, selected from the participants of the 2014, 2016, 2018, 2020 National Health and Nutrition Examination Survey. Our questions included self-perceived health status, physical activity (aerobic exercise, muscle strengthening exercise, walking and sedentary time), and mental health (depression, stress). The statistical analysis was performed using SAS 9.4 (SAS Institute Inc, Cary, NC, USA), and a complex sample analysis was performed using weights according to the KNHANES raw data usage guidelines from the Korea Centers for Disease Control and Prevention. The results of the data analyses showed that cancer survivors with self-perceived good health showed eight times lower levels of stress and five times lower levels of depression. In addition, the stress of cancer survivors with self-perceived good health was measured as about two times lower during the walking exercise. The depression index was measured as lower in the case of the walking exercise than in the case of the non-walking exercise. In conclusion, to manage depression and stress in cancer survivors, it is recommended to regularly monitor their subjective health condition, encourage positive evaluations of their health, and suggest continued participation in activities such as walking.

## 1. Introduction

In 2020, the incidence of cancer per 100,000 persons in South Korea was 496.2, and the probability of cancer at the end-of-life expectancy was 36.9% [1]. In addition, with the advancement of cancer diagnosis and treatment techniques, the five-year relative survival in cancer patients from 2016 to 2020 was 71.5%, indicating that seven or more out of 10 cancer patients lived beyond five years [1]. In the past decade, the rate of cancer survival in South Korea has markedly increased, which has shifted the focus regarding cancer survivors from ‘how long to live’ to ‘what kind of life they will live while maintaining health’. As a consequence, interest in the health-related quality of life of cancer patients is increasing. Health-related quality of life is ‘physical and mental health perceived by an individual or group over time’ [2], and is composed of self-perceived health, physical function, and psychological well-being [3]. Therefore, it can be said that the management of related factors is essential to improve the health-related quality of life of cancer survivors.

Cancer survivors endure physical as well as mental pain from the diagnosis of cancer and throughout subsequent processes, such as surgery, chemotherapy, and radiation therapy [4]. The psychological stress leads to depression and in fact, the prevalence of depression in cancer patients is approximately 20%, a three-times higher level than the general population [5]. Stress and depression in cancer patients negatively affect cancer progression, treatment process, and prognosis. Additionally, these factors can worsen the quality of life for both patients and their families [6]. Hence, mental health, including stress and depression, is a major issue in cancer survivors that demands attention, and efforts to improve mental health are needed.

Physical activity is a safe, feasible, and relatively inexpensive, non-pharmacological means to improve mental health. Therefore, it is one of the first recommended methods for managing depression in cancer survivors [7]. For this reason, physical activity has recently been recommended to cancer survivors as soon as possible after cancer diagnosis [8].

While treating the chronic disease called cancer, patients lose confidence in their health due to damage to their body functions and many complications [9]. Hence, self-perceived health is bound to decrease with the fall in physical fitness during the process of cancer treatment. In the process of adapting to these changes, poor health, expressed as self-perception of health, causes stress and increased depression [10].

Therefore, the purpose of this study was to present basic data for improving the health-related quality of life of cancer survivors by investigating the relationship between self-perceived health and physical activity as it relates to the mental health of cancer survivors.

## 2. Materials and Methods

### 2.1. Study Design

In this study, a secondary data analysis was performed using data from the 2014, 2016, 2018, 2020 Korea National Health and Nutrition Examination Survey (KNHANES), a statutory survey conducted by the Korea Centers for Disease Control and Prevention on about 10,000 people (https://knhanes.kdca.go.kr/knhanes, accessed on 20 August 2022). This study was described in accordance with the Strengthening the Reporting of Observational Studies in Epidemiology (STROBE) guidelines (https://www.strobe-statement.org/index.php?id=strobe-home (accessed on 4 November 2022)).

### 2.2. Participants and Data Collection

Among the 31,051 participants in the 2014, 2016, 2018, 2020 KNHANES data, the number of adults aged ≥19 years with a past diagnosis of cancer was 1144. Among them, 718 were cancer survivors for more than 5 years, with the exclusion of those who had not responded to the questions regarding self-perceived health (no missing), physical activity (n = 22), stress (n = 4) and depression (n = 16), 688 participants were selected for the analyses.

### 2.3. Instruments

#### 2.3.1. General Characteristics

General characteristics, such as marital status (non-married married, marred but single), income levels (Q1, Q2, Q3, Q4), economic activities (yes or no), and education levels (elementary school, Middle school, Hight school, Collage) were used.

#### 2.3.2. Self-Perceived Health

Self-perceived health was measured using the question *‘How do you perceive your general state of health?’*, which was rated on a 5-point Likert scale; ‘Very Good’, ‘Good’, ‘Moderate’, ‘Poor’ and ‘Very Poor’. Three groups were formed: ‘*Good*’, combining ‘Very Good’ and ‘Good’; ‘*Moderate*’, indicating ‘Moderate’; ‘*Poor*’, combining ‘Very Poor’ and ‘Poor’.

#### 2.3.3. Physical Activity

Physical activity was measured based on aerobic exercise, muscle-strengthening exercise, walking and sedentary time. Aerobic exercise was defined as at least 2 h 30 min moderate-intensity exercise, at least 1 h 15 min high-intensity exercise, or the equivalent time of combination of moderate- and high-intensity exercises (1 min high-intensity exercise as equivalent to 2 min moderate-intensity exercise). Muscle-strengthening exercise was defined as two or more days in the past week of performing an exercise related to muscle strengthening such as press-ups, sit-ups, dumbbell lifting, weight lifting and bar exercise. Walking was defined as at least five days of performing 30 min or more walking in the past week. Sedentary time was defined as at least six hours of sitting in total. using the question ‘*How many hours a day do you spend sitting or lying down somewhere?’* [11].

#### 2.3.4. Mental Health

Mental health was measured using questions in the depression screening tool (Patient Health Questionnaire 9 (PHQ-9)) [12] and in those of the stress indicators. The PHQ-9 consisted of nine questions on how often the participant suffered from a given symptom in the past two weeks, where each question was rated on a scale of 0–3: Not at all (0), ‘Several days’ (1), ‘A week or more’ (2) and ‘Almost every day’ (3), with the total score of 27. The PHQ-9 score was the sum of the scores of questions 1–9, and higher scores indicated poorer states of mental health [12].

Stress was measured based on the choice of response to the question *‘How would you rate your level of stress in daily life?’* Using the responses, the participants were grouped into two levels of perceived stress. The high-stress group contained those whose responses were ‘Very much’ or ‘Much’ to rate their stress in daily life and the low-stress group contained those whose responses were ‘Little’ or ‘Almost none’.

### 2.4. Data Analysis

For statistical analysis, SAS ver. 9.4 (SAS Institute Inc, Cary, NC, USA) was used. Following the guideline of raw data use suggested by the KNHANES of KDCA, weighted values were used to perform complex sampling. The distribution of self-perceived health, characteristics related to physical activity, and levels of stress and depression in cancer survivors were analyzed based on their general characteristics. Subsequently, the distribution of stress and mean differences in depression were analyzed based on self-perceived health and physical activity-related characteristics. We utilize the Rao–Scott Chi-square test for analyzing categorical variables, and the general linear model (GLM) for analyzing continuous variables.

### 2.5. Ethical Considerations

This study represents a secondary analysis of national survey data available through the Korea National Health and Nutrition Examination Survey website and was conducted after receiving approval from the institutional review board of K University [KYUHIRB-2023-02-019]. Because the raw data analyzed for this study contained no personally identifiable information, anonymity and confidentiality were guaranteed.

## 3. Results

### 3.1. Variation in Self-Perceived Health According to General Characteristics

The subjects consisted of 68.6% females and 31.4% males. The mean age was 60.5 years (SE = 0.53) and the individuals aged ≥ 50 years accounted for 82.3%. For marital status, 97.7% were married (79.2% living with spouse and 18.5% without spouse). For household income, the highest level at 27.4% was shown by Q4, followed by Q3 25.5%, Q1 24.6% and Q2 22.5%. The individuals currently engaged in an employment accounted for 48.5% and more than half of the participants (60.7%) had a high school degree. Females (35.6%) had a higher percentage of respondents with “poor” self-perceived health than males (23.5%). Unmarried individuals showed the highest percentage of respondents with ‘poor’ self-perceived health at 52.8%, followed by married but living without spouse (29.9%) and married and living with spouse (37.2%). Q1 showed the highest percentage of respondents with ‘poor’ self-perceived health at 46.0%, while the difference between the individuals without employment (36.9%) and those with current employment (26.5%) was 10.4% p. Those who had graduated from elementary school showed the highest percentage of respondents with ‘poor’ self-perceived health at 44.8% (Table 1).

**Figure 1:**
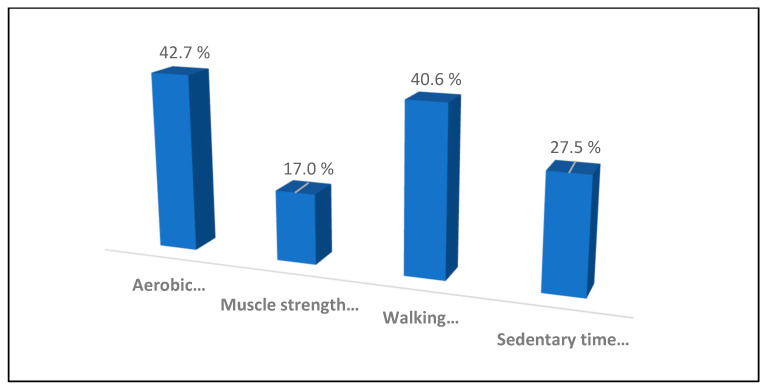
Percentage of physical activity.

Among the cancer survivors, 42.7% did aerobic exercise, 17.0% did muscle-strengthening exercise, 40.6% did walking and 27.5% had sedentary time below six (Figure 1).

Analyzing the variations in physical activity according to general characteristics, there was a difference in the level of strength exercise by gender (27.1% male, 12.4% female). Depending on age, there was a difference in aerobic exercise, strength exercise, and walking. Aerobic exercise was the highest at 40–49 years old (54.1%), strength exercise was 23.1% at 60–69 years old, and walking was 45.2% at 50–59 years old. Depending on marital status, there was a difference in aerobic exercise and walking, and the level of physical activity of married people with a spouse was high, at 45.6% and 42.8%, respectively. There was a difference in aerobic exercise according to household income, and the aerobic exercise practice rate increased as household income increased. Depending on the level of education, there were differences in aerobic exercise, strength training, and sedentary time, and when the level of education was high, the level of physical activity was high, and the rate of sedentary time for less than six hours was also high (Table 2).

In the subsequent analysis of the variation in stress according to general characteristics, significant differences were found for the gender, age and education. The percentage of individuals with a high level of stress was higher in females (31.4%) than in males (68.6%), showing a 37.2%p difference, and the largest proportion of cancer survivors (31.4%) belonged to 50–59 years in age group. And in middle school graduates, the percentage of individuals with high stress was the lowest at 12.9%.

The depression score was ranged 0~24 and mean was 2.83 (SE = 0.19). The top 10% of cancer survivors had mild depression and the top 5% had a moderate or higher level of depression. When depression scores were analyzed according to general characteristics, significant differences were found between sex, marital status, household income and education levels: females had a higher mean score (3.15), and the highest mean score was shown by unmarried individuals (6.32) and cancer survivors belonging to Q1 (4.3). The lower the level of education, the higher the mean score (Table 3).

### 3.2. Mental Health According to Self-Perceived Health, Physical Activity

The respondents with ‘poor’ self-perceived health (5.5%) had a stress level that was sight times higher than who ‘good’ self-perceived health (42.0%). Depression scores were higher in the respondents with ‘poor’ self-perceived health (5.26 on mean) than in respondents with ‘good’ self-perceived health (0.77 on mean).

The state of mental health varied significantly according to the level of physical activity (walking). The percentage of cancer survivors with a high level of stress was about two times higher in those without walking (28.0%) than in those with walking (15.2%). Depression varied significantly according to walking. The mean depression score was 1.89 in cancer survivors with walking but 3.45 in those without walking, which suggests that walking could reduce depression (Table 4).

## 4. Discussion

Cancer survival is a dynamic process that continues throughout one’s life as it moves on from cancer diagnosis to complete cure. In this process, cancer survivors experience various physical and psychological symptoms and social problems, as well as aftermaths [13]. Thus, this study investigated the relationship between self-perceived health and physical activity in cancer survivors’ mental health in order to improve their health-related quality of life.

Cancer survivors with a good self-perception of health showed an eight times lower level of stress and five times lower level of depression than those with a poor self-perception of health. Survivors experience significant stress and depression throughout the entire process from cancer diagnosis to treatment [14,15,16]. In addition, various medical procedures from surgery to chemotherapy and radiotherapy unavoidably induce physical changes in patients, as a result of which the self-perceived health of the patient becomes poor [17]. However, patients themselves, as well as their families and healthcare staff, tend to regard stress and depression related to cancer diagnosis as inevitable and, therefore, not noteworthy, while the presumption that depression cannot be lifted until the complete cure of cancer leads to passive attitudes towards diagnosis and treatment [18]. Stress and depression have been identified as predictors of cancer mortality [19], and suggested as factors that shorten the survival periods of cancer patients [20]. Thus, thorough assessments of self-perceived health in cancer survivors should be conducted continuously from the early days of cancer diagnosis. To ensure that cancer patients positively rate their subjective health, intervention programs should be developed through the support system of family and healthcare staff. In addition, continuous research is needed on the effects of subjective health status as perceived by patients, and it is necessary for healthcare providers to prepare intervention plans to frequently assess and manage stress and depression in all cancer patients.

The percentage of cancer survivors with high levels of stress was two times lower when they performed walking exercises than when they were not physically active. The depression index was measured lower in the case of those who performed walking exercises than in the cases of those who did not perform walking exercises. Previous studies have shown that physical activity is effective not only in reducing psychological stress such as anxiety and depression [21], but also in preventing cancer, secondary cancer, and recurrence, and in increasing survival rates [22]. Notably, physical activity is the representative non-pharmacological method of managing stress and depression in cancer survivors, which is safe and practical and has a relatively low cost, and has been the recommended method [23]. Physical activity is generally known to increase the firing of serotonin neurons, facilitating the release and synthesis of serotonin to improve mood and reduce depressive symptoms [24]. A low level of serotonin is associated not only with depression but also with chronic pain, and the implications of such complex mechanisms can partially account for the results of this study, where the levels of stress and depression in cancer survivors were lower in those performing walking or muscle-strengthening exercise. A previous study showed that a shared finding is that depressive symptoms could be reduced by up to 50% or higher through exercise and physical activity, based on which it is recommended that an intervention of exercise be given to cancer patients as soon after cancer diagnosis as possible to lower depressive symptoms [23]. Further studies should also be conducted regarding numerous factors associated with exercise so as to effectively reduce rates of depression. Additionally, clear, and consistent evidence on physical activity in cancer survivors in South Korea should be suggested through repeated studies and randomized studies, as there are currently no clear guidelines on physical activity for cancer survivors in South Korea, while guidelines in the U.S. recommend a moderate or higher level of physical activity for cancer survivors [9].

This study provides a cross-sectional examination of the factors associated with health-related quality of life, which include self-perceived health, physical activity and mental health among cancer survivors using a national representative sample. However, as with many other studies, this study has its limitations, such that the results should be interpreted with consideration of its design. First, this study has a limitation in that a causal relationship is unclear as it is a cross-sectional study by nature. Second, questions may arise regarding differences in treatment toxicity, economic burden, and lifetime prognosis depending on the type of cancer in the study subjects. Third, we did not consider the type of cancer diagnosed or treatment received that could affect self-perceived quality of life. Finally, since this study used a single item to measure stress, a large-scale study to overcome this limitation should be conducted in the future.

## 5. Conclusions

As cancer is recognized as a chronic disease according to studies showing an increase in cancer survival rates, the importance of quality of life along with continuous healthcare for cancer survivors is now being highlighted. In light of this, the purpose of this study was to investigate the relationship between self-perceived health and physical activity on the mental health of cancer survivors, which are factors related to health-related quality of life. The results demonstrated that people who rated their self-perceived health as good had lower levels of stress and depression. In addition, the percentage of cancer survivors with a high level of stress was low in those performing walking or muscle-strengthening exercise, and the level of depression was low in those performing walking. To conclude, for the management of stress and depression in cancer survivors, continuous monitoring of self-perceived health should be conducted to assist with the positive rating of subjective health in cancer patients, who are recommended to maintain steady levels of walking or muscle-strengthening exercise. This study is significant in that it provides basic data for improving the health-related quality of life of cancer survivors by identifying the relationship between self-perceived health and physical activity on the mental health of cancer survivors.

## Figures and Tables

**Table 1:** Variation in self-perceived health according to general characteristics.

		Good		Moderate		Poor		Total		χ^2^	*p*
		n	%	n	%	n	%	n	%		
Sex	Male	51	24.6	113	51.9	60	23.5	224	31.4	7.244	0.027
	Female	75	19.9	218	44.5	171	35.6	464	68.6		
	Total	126	21.4	331	46.8	231	31.8	688	100.0		
Age	19–39 year	6	27.7	11	38.2	6	34.1	23	4.6	10.284	0.246
	40–49 year	21	32.1	31	47.6	19	20.3	71	13.1		
	50–59 year	25	18.8	88	46.2	54	35.0	167	31.4		
	60–69 year	29	20.2	99	52.1	58	27.7	186	24.5		
	>70 year	45	19.1	102	43.8	94	37.1	241	26.3		
	Total	126	21.4	331	46.8	231	31.8	688	100.0		
Marital status	Unmarried	1	3.4	7	43.8	5	52.8	13	2.3	9.717	0.046
	Married (with spouse)	108	23.6	253	46.4	168	29.9	529	79.2		
	Married (without spouse)	17	13.9	71	49.0	58	37.2	146	18.5		
	Total	126	21.4	331	46.8	231	31.8	688	100.0		
Household income	Q1	19	10.5	79	43.4	83	46.0	181	24.6	28.850	<0.001
	Q2	20	16.8	78	47.4	62	35.8	160	22.5		
	Q3	41	29.1	85	48.1	46	22.8	172	25.5		
	Q4	46	28.0	88	47.6	39	24.4	173	27.4		
	Total	126	21.5	330	46.7	230	31.9	686	100.0		
Employment	Yes	60	22.6	157	50.9	85	26.5	302	48.5	6.115	0.047
	No	66	20.3	173	42.8	146	36.9	385	51.5		
	Total	126	21.4	330	46.7	231	31.9	687	100.0		
Education level	Elementary school	24	12.8	98	42.4	106	44.8	228	26.4	32.561	<0.001
	Middle school	8	8.9	50	51.2	40	40.0	98	12.9		
	High school	48	25.8	110	50.6	50	23.6	208	34.4		
	University	46	30.2	73	44.3	35	25.5	154	26.3		
	Total	126	21.4	331	46.8	231	31.8	688	100.0		

**Table 2:** Physical activity according to general characteristics.

		Aerobic (YES)			Muscle Strength (YES)			Walking (YES)			Sedentary Time (<6 h)		
		n	%	*p*	n	%	*p*	n	%	*p*	n	%	*p*
Sex	Male	104	48.1	0.095	60	27.1	<0.001	97	43.9	0.302	166	75.9	0.243
	Female	180	40.3		59	12.4		176	39.1		324	71.0	
Age	19–29 year	8	46.9	0.021	5	19.7	0.046	3	9.6	0.016	16	80.2	0.195
	40–49 year	26	54.1		12	20.1		26	34.3		52	72.2	
	50–59 year	41	46.4		19	9.9		77	45.2		115	71.9	
	60–69 year	44	49.2		40	23.1		76	44.0		123	66.0	
	>70 year	23	23.2		43	17.7		91	40.5		184	78.2	
Marital status	Unmarried	5	32.9	0.043	5	33.4	0.092	3	12.2	0.035	11	93.5	0.070
	Married (with spouse)	236	45.6		93	17.8		225	42.8		365	71.1	
	Married (without spouse)	43	31.6		21	11.3		45	34.8		114	76.0	
Household income	Q1	67	36.1	0.038	28	16.1	0.817	68	39.5	0.189	123	71.6	0.353
	Q2	54	35.9		24	14.5		59	38.5		108	66.6	
	Q3	76	47.0		31	16.6		63	36.1		133	74.9	
	Q4	87	51.1		35	19.0		83	48.1		124	75.6	
Employment	Yes	136	46.1	0.102	54	16.3	0.707	111	36.3	0.106	194	68.6	0.053
	No	147	39.4		64	17.4		161	44.4		295	76.2	
Education level	Elementary school	66	27.6	<0.001	28	13.6	0.038	81	39.6	0.089	161	70.1	0.027
	Middle school	39	38.5		13	11.2		35	31.4		67	73.2	
	High school	97	48.5		39	16.3		96	47.4		140	67.3	
	University	82	52.4		39	24.2		61	37.1		122	81.6	

**Table 3:** Stress and depression according to general characteristics.

		Stress (Yes)				Depression			
		n	%	χ^2^	*p*	Mean	SE	F	*p*
Sex	Male	224	31.4	9.659	0.004	2.08	0.24	9.830	0.002
	Female	464	68.6			3.15	0.26		
Age	19–29 year	23	4.6	4.799	0.047	5.37	1.78	0.840	0.502
	40–49 year	71	13.1			2.32	0.45		
	50–59 year	167	31.4			2.90	0.32		
	60–69 year	186	24.5			2.59	0.37		
	>70 year	241	26.3			2.73	0.30		
Marital status	Unmarried	13	2.3	6.258	0.091	3.13	0.08	122.650	<0.001
	Married (with spouse)	529	79.2			2.18	0.04		
	Married (without spouse)	146	18.5			3.56	0.11		
Household income	Q1	181	24.6	1.041	0.100	4.28	0.48	8.210	<0.001
	Q2	160	22.5			2.97	0.36		
	Q3	172	25.5			1.93	0.24		
	Q4	173	27.4			1.97	0.26		
Employment	Yes	302	48.5	8.932	0.308	2.53	0.24	2.150	0.143
	No	385	51.5			3.09	0.30		
Education level	Elementary school	228	26.4	8.167	0.030	3.81	0.40	5.700	0.001
	Middle school	98	12.9			3.78	0.77		
	High school	208	34.4		.	2.36	0.26		
	University	154	26.3			1.95	0.28		

**Table 4:** Mental health according to self-perceived health, physical activity.

			Stress (Yes)				Depression			
			n	%	χ^2^	*p*	Mean	SE	F	*p*
Self-perceived health		Good	8	5.5	66.190	<0.001	0.77	0.14	57.810	<0.001
		Moderate	63	17.7			2.09	0.23		
		Poor	87	42.0			5.26	0.41		
Physical activity	Aerobic	no	101	24.9	1.516	0.218	3.12	0.29	3.550	0.060
		yes	57	20.1			2.41	0.24		
	Muscle strength	no	135	23.2	0.174	0.676	2.80	0.21	0.040	0.851
		yes	23	21.1			2.91	0.55		
	Walking	no	116	28.0	8.035	<0.001	3.45	0.28	19.87	<0.001
		yes	42	15.2			1.89	0.22		
	Sedentary time	<6 h	45	21.5	0.205	0.651	2.53	0.27	1.210	0.271
		>6.1 h	113	23.3			2.93	0.25		

## Data Availability

Not applicable.
